# Adaptive Trajectory-Constrained Heading Estimation for Tractor GNSS/SINS Integrated Navigation

**DOI:** 10.3390/s26020595

**Published:** 2026-01-15

**Authors:** Shupeng Hu, Song Chen, Lihui Wang, Zhijun Meng, Weiqiang Fu, Yaxin Ren, Cunjun Li, Hao Wang

**Affiliations:** 1 School of Instrument Science and Engineering, Southeast University, Nanjing 210096, China; 2 State Key Laboratory of Intelligent Agricultural Power Equipment, Luoyang 471039, China; lycs1974@126.com; 3 Research Center of Intelligent Equipment, Beijing Academy of Agriculture and Forestry Sciences, Beijing 100097, Chinafuwq@nercita.org.cn (W.F.); 4 Information Technology Research Center, Beijing Academy of Agriculture and Forestry Sciences, Beijing 100097, China; renyx@nercita.org.cn (Y.R.); licj@nercita.org.cn (C.L.)

**Keywords:** autonomous navigation, sensor fusion, inertial navigation system, heading measurement, adaptive algorithm, trajectory constraint, robot tractor

## Abstract

**Highlights:**

**What are the main findings?**

- An adaptive trajectory-constrained heading estimation method using SWAEKF is proposed, achieving rapid convergence (<10 s for straight lines) and high accuracy (RMS heading error <0.15°) in low-speed tractor navigation.
- A 23% improvement in heading accuracy and 62% reduction in convergence time compared to conventional adaptive EKF are demonstrated through field tests, including straight and curved paths.

**What are the implications of the main findings?**

- A cost-effective solution is provided for the autonomous navigation of small-to-medium tractors by enabling precise heading estimation with single-antenna GNSS/SINS integration, reducing reliance on dual-antenna systems.
- Adaptability to low-dynamic farmland environments is enhanced, supporting the advancement of agricultural automation with robust sensor fusion.

**Abstract:**

Accurate heading estimation is crucial for the autonomous navigation of small-to-medium tractors. While dual-antenna GNSS systems offer precision, they face installation and safety challenges. Single-antenna GNSS integrated with a low-cost Strapdown Inertial Navigation System (SINS) presents a more adaptable solution but suffers from slow convergence and low accuracy of heading estimation in low-speed farmland operations. This study proposes an adaptive trajectory-constrained heading estimation method. A sliding-window adaptive extended Kalman filter (SWAEKF) was developed, incorporating a heading constraint model that utilizes the GNSS-derived trajectory angle. An enhanced Sage–Husa algorithm was employed for the adaptive estimation of the trajectory angle measurement variance. Furthermore, a covariance initialization strategy based on the variance of trajectory angle increments was implemented to accelerate convergence. Field tests demonstrated that the proposed method achieved rapid heading convergence (less than 10 s for straight lines and 14 s for curves) and high accuracy (RMS heading error below 0.15° for straight-line tracking and 0.25° for curved paths). Compared to a conventional adaptive EKF, the SWAEKF improved accuracy by 23% and reduced convergence time by 62%. The proposed algorithm effectively enhances the performance of GNSS/SINS integrated navigation for tractors in low-dynamic environments, meeting the requirements for autonomous navigation systems.

## 1. Introduction

Global Navigation Satellite System (GNSS) technology offers a key solution for real-time centimeter-level positioning. Specifically, the rapid and accurate direction-finding capability of dual-antenna GNSS receivers enhances the applicability of autonomous tractor navigation [1]. In recent years, with the growing demand for automation in small and medium horsepower tractors, the use of dual-antenna GNSS in navigation systems has encountered challenges, such as potential safety hazards due to an overly wide baseline and poor installation adaptability [2]. While autonomous agricultural machinery systems based on dual-antenna GNSS have been widely applied with significant outcomes [3], the integration of a single-antenna GNSS with a low-cost microelectromechanical system (MEMS) inertial measurement unit (IMU) through multi-sensor fusion demonstrates superior adaptability [4]. Relevant research shows that when the heading measurement accuracy is less than 0.5°, it can meet the requirements of automatic control for agricultural machinery navigation [5]. Consequently, integrated devices combining single-antenna GNSS and IMU have emerged as a research focus in tractor autonomous navigation, where achieving precise heading measurement through fusion represents the key technical challenge.

To achieve heading alignment for GNSS/INS integrated navigation, extensive research has been conducted by scholars, including methods such as fast and accurate stationary gyro compassing and dynamic coarse measurement for moving bases [6]. However, for low-cost MEMS-IMU, heading measurement algorithms are primarily categorized into two types. One approach formulates the measurement as an optimization problem. For instance, a novel fast indirect in-motion coarse measurement model and an adaptive Student’s t-based Kalman filter (STKF) have been proposed; this method utilizes GPS speed and rotation vectors as the measurement model and SINS attitude parameterization for the process model, with car-mounted simulations and experiments showing a heading error below 0.31° even in hostile environments [7]. An innovative in-motion heading alignment method utilizing time-differenced carrier phases (TDCP) from a single GNSS antenna and a low-grade IMU based on the fundamental principle of trajectory similarity, the experimental results show that the heading could be determined with an accuracy of 0.49° at a 95% confidence level for the land vehicle, and the RMS of the alignment errors is 0.24° [8]. Another new method combines a modified double-vectors construction approach with a fast IFA method based on gradient descent, avoiding complex matrix operations; vehicle experiments demonstrated a reduction in heading error from 2.69° to 1.41° and alignment time from 38.79 s to 6.08 s compared to the QUEST algorithm [9]. However, these optimization-based alignment (OBA) methods require the vehicle to perform sufficient maneuvering with acceleration changes for optimal performance, making them unsuitable for low-speed scenarios and low-grade SINS.

The second category of methods focuses on constraining the state space by leveraging the specific motion characteristics of the vehicle carrier. For instance, to achieve rapid heading initialization for tilted Real-Time Kinematic (RTK) receivers, a technique correcting the IMU heading using the horizontal direction of the RTK position vector was developed, capitalizing on the short-term trajectory similarity between INS and GNSS. This method achieved a heading initialization accuracy of 1.15° within 2–3 s [10]. Similarly, in low-speed scenarios, integrated INS/GNSS systems have utilized the vector directions from consecutive GNSS trajectory points and INS dead reckoning (DR) points to correct the gyroscope-derived heading, enabling rapid initialization [11]. Building on the principle of trajectory similarity, another approach leveraged real-time GNSS carrier-phase observations to assist INS heading convergence, reporting heading accuracies of 0.68° and 1.68° within 5 s in open-sky and typical urban environments, respectively [12,13]. Furthermore, the use of virtual velocity constructed from Non-Holonomic Constraints (NHC) as Kalman Filter observations has shown promising results, with one study achieving a measurement azimuth accuracy of 0.0358° (RMS) [14]. Advancements include reconstructing error models in Lie group space combined with left-invariant Kalman filtering to aid GNSS/SINS integration based on NHC, yielding a heading accuracy of 0.1° in passenger car tests conducted in open urban road environments [15]. Another approach involved constructing an EKF to estimate ground heading, velocity, and heading rate states using GNSS latitude and longitude. While this method performed well on an Unmanned Surface Vehicle (USV) at speeds above 1 m/s, its accuracy significantly decreased at lower speeds [16]. Some researchers have also explored velocity-aided optimization-based methods using least-square estimation with GNSS velocity assistance. A tractor test using an industrial-grade MEMS IMU demonstrated that this method could reduce heading measurement errors to approximately 4° within 60 s at a speed of about 1 m/s [17]. Additionally, a fusion algorithm incorporating fuzzy adaptive rules and a forgetting factor operator for quaternion-based attitude calculation, integrating attitude angles with GNSS heading angles, was proposed for tractor attitude measurement. Experimental results indicated a significant reduction in the tractor’s position error after attitude angle correction [18]. However, a common limitation of many constraint-based methods is their reliance on the vehicle satisfying the non-holonomic constraints typical of ground-wheeled vehicles. Furthermore, the accuracy of these methods is often intrinsically linked to the vehicle’s speed. Consequently, existing research results still struggle to meet the demanding requirements for fast convergence and sustained accuracy under the continuous low-speed and non-maneuvering conditions characteristic of tractor automatic navigation operations.

To tackle the persistent challenges of slow heading error convergence and low heading accuracy during low-speed tractor operations in farmland, this study proposes a novel heading measurement method based on GNSS trajectory constraints, integrated with a Sliding-window Adaptive Extended Kalman Filter (SWAEKF). This method falls within the category of constraining the carrier’s motion characteristics in the state space. Through strategic algorithm model optimization, the method aims to achieve rapid convergence of heading errors and enhance dynamic heading accuracy. The principal contributions of this work are summarized as follows:

(1) A novel heading constraint model that switches between static and dynamic observations based on tractor velocity thresholds, improving robustness during low-speed operations.
(2) An enhanced Sage–Husa adaptive algorithm that estimates trajectory angle measurement variance using a sliding-window of innovation sequences, mitigating the impact of GNSS noise.
(3) A covariance initialization strategy for the heading error angle based on the statistical variance of initial trajectory increments, accelerating convergence.
(4) Comprehensive validation through field tests covering straight-line, curved-path, and automated navigation scenarios, demonstrating superior performance compared to conventional methods.

## 2. Materials and Methods

### 2.1. System Framework and Experimental Platform

**Figure 1:**
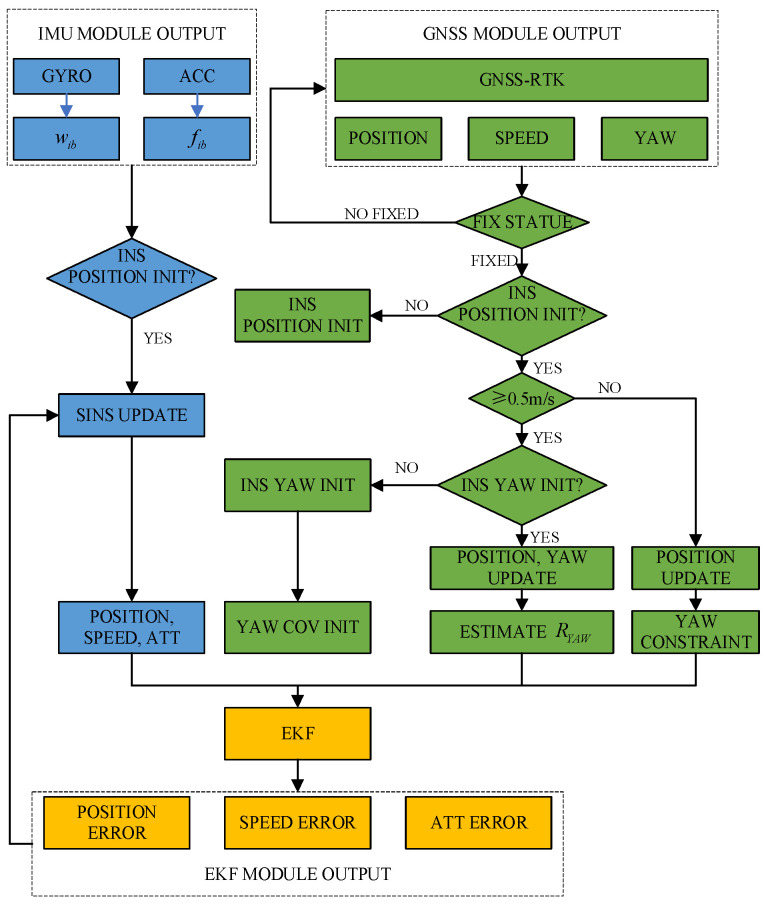
Heading measurement framework and system implementation flowchart.

**Figure 2:**
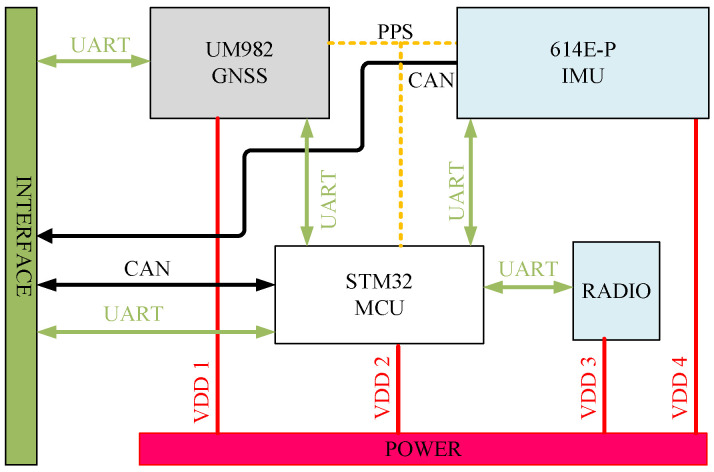
Architecture of hardware diagram.

The heading measurement system integrates a single-antenna GNSS receiver (Z301 Pos, Ver. V1.1.2, Nongxin Science & Technology (Beijing) Co., Ltd., Beijing, China) and a low-cost MEMS IMU (FSS-IMU614E-P, Ver. 12789, Forsense (Shanghai) Technology Co., Ltd., Shanghai, China) mounted on a 704 tractor (LOVOL Heavy Industry Co., Ltd., Weifang, China). The system architecture, illustrated in Figure 1, synchronizes GNSS data (10 Hz) and IMU data (100 Hz) via a CAN bus, with a dual-antenna GNSS receiver (Z202, Ver. V1.3.7, Nongxin Science & Technology (Beijing) Co., Ltd., Beijing, China) serving as the heading reference. The hardware architecture of the Z301 Pos device is shown in Figure 2, where the radio module (Harxon Co., Ltd., Shenzhen, China) receives RTCM data from the base station and forwards it to the GNSS module via an MCU. The GNSS module processes satellite signals and RTCM data to achieve RTK positioning, while the MCU collects and packages IMU and GNSS data for output.

**Figure 3:**
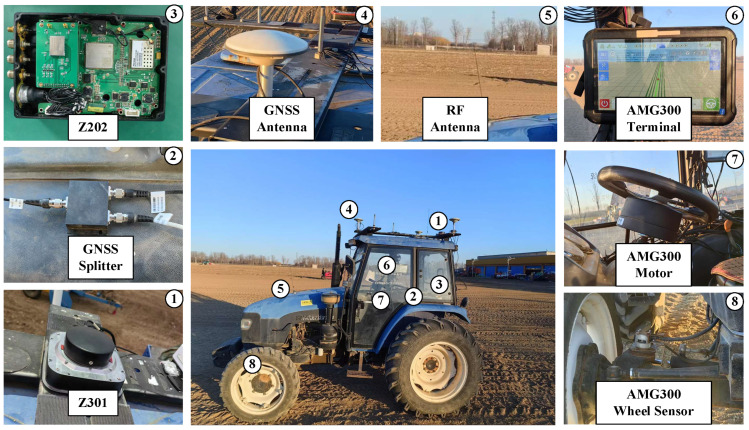
Composition diagram of the experimental platform.

**Figure 4:**
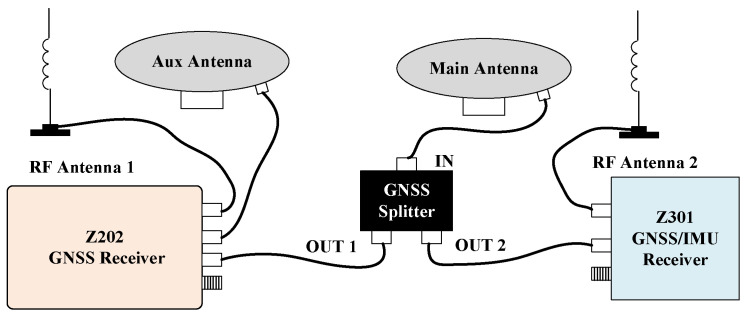
Device connection diagram.

**Figure 5:**
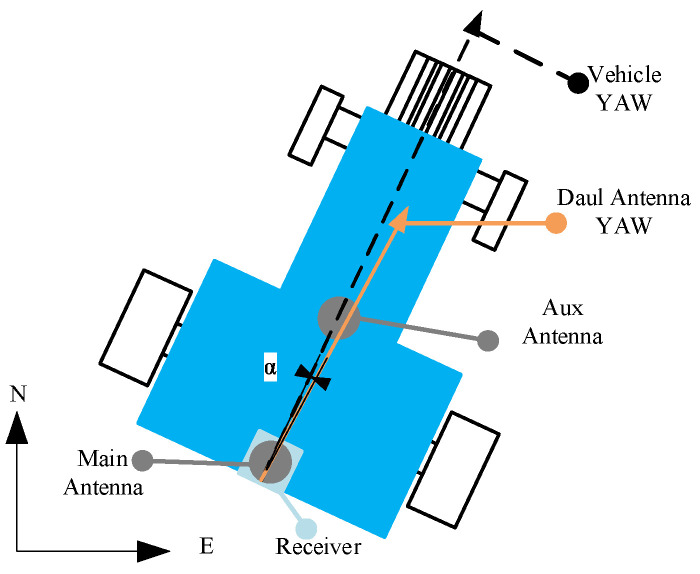
Antenna configuration diagram of Z202 receiver.

Key hardware specifications are summarized in Table 1. The experimental platform, depicted in Figure 3, includes an AMG300 (Ver. 3.31.102.3, Nongxin Science & Technology (Beijing) Co., Ltd., Beijing, China) autosteering system for path control, ensuring repeatability in field tests. The device connections are detailed in Figure 4, where the Z202 receiver shares the positioning antenna with the Z301 Pos via a GNSS splitter (Beijing East Sheep Science & Technology Co., Ltd., Beijing, China). The antenna (Harxon Co., Ltd., Shenzhen, China) configuration of the Z202 receiver is shown in Figure 5, with the baseline direction calibrated to correct installation errors.

### 2.2. GNSS/IMU Data Fusion Based on EKF

The fusion of GNSS and IMU data involves two primary components: the SINS mechanical arrangement and the integrated navigation algorithm [19,20]. The differential equation governing the update of the SINS mechanical arrangement is given by:

(1) $$\left\{\begin{array}{l} {\dot{C}}_{b}^{n}=C_{b}^{n}(\omega_{ib}^{b}\times )-\left[\left(\omega_{ie}^{n}+\omega_{en}^{n}\right)\times \right]C_{b}^{n}\\ \dot{v}=C_{b}^{n}f_{\mathrm{sf}}^{b}-\left(2\omega_{ie}^{n}+\omega_{en}^{n}\right)\times v+g\\ \dot{p}=M_{pv}v \end{array}\right.$$

where ${\dot{C}}_{b}^{n}$ is the differential of attitude matrix from the b-frame to the n-frame; $C_{b}^{n}$ represents the attitude matrix; $\omega_{ib}^{b}$ represents the angular rate vector of gyro; $\omega_{ie}^{n}$ represents the Earth’s rotation rate; $\omega_{en}^{n}$ represents the angular rate vector of the n-frame relative to the e-frame; $\dot{v}$ is the differential of velocity vector; v represents the velocity vector; $f_{\mathrm{sf}}^{b}$ represents the specific force vector of accelerometer; g is the gravity acceleration vector; $\dot{p}$ is the differential of position vector; $M_{pv}$ represents the transfer matrices of velocity with the derivative of position.

Considering the measurement errors of the IMU and the initialization errors of attitude, orientation, velocity, and position, all of which will propagate through the update algorithm, leading to the continuous accumulation of errors in the SINS. The error equation for SINS is analyzed as follows:

(2) $$\left\{\begin{array}{l} \dot{\phi }=M_{aa}\phi +M_{av}\delta v+M_{ap}\delta p-C_{b}^{n}\varepsilon \\ \delta \dot{v}=M_{va}\phi +M_{vv}\delta v+M_{vp}\delta p+C_{b}^{n}𝛻\\ \delta \dot{p}=M_{pv}\delta v+M_{pp}\delta p \end{array}\right.$$

where $\dot{\phi }$ represents the derivative of attitude error; ϕ represents the attitude error; $M_{aa}$, $M_{av}$, $M_{ap}$, respectively, represent the transfer matrices that describe the relationship between the attitude error, velocity error, position error and the differential of attitude error; δv represents the velocity error; δp represents the position error; ε represents the gyro bias; $\delta \dot{v}$ represents the derivative of velocity error; $M_{va}$, $M_{vv}$, $M_{vp}$, respectively, represent the transfer matrices that describe the relationship between the attitude error, velocity error, position error and the differential of velocity error; $\delta \dot{p}$ represents the derivative of position error; 𝛻 represents the accelerometer bias; $M_{pp}$ represents the transfer matrices of position errors with the derivative of position error.

To accurately estimate the SINS errors, the attitude error ϕ, velocity error δv, position error δp, gyro bias ε, and accelerometer bias 𝛻 are selected as the 15-dimensional state vector X, as shown below:

(3) $$X={\left[\begin{array}{c} \phi^{T}\;{\left(\delta v\right)}^{T}\;{\left(\delta p\right)}^{T}\;\begin{array}{cc} {\left(\varepsilon \right)}^{T} & {\left(𝛻\right)}^{T} \end{array} \end{array}\right]}^{T}$$

Based on the Equation (2), simplified system noise matrix, the state-space model for the integrated navigation algorithm is established as follows:

(4) $$\left\{\begin{array}{l} \dot{X}=FX+W\\ Z=HX+V \end{array}\right.$$

(5) $$F=\left[\begin{array}{ccccc} M_{aa} & M_{av} & M_{ap} & -C_{b}^{n} & 0_{3\times 3}\\ M_{va} & M_{vv} & M_{vp} & 0_{3\times 3} & C_{b}^{n}\\ 0_{3\times 3} & M_{pv} & M_{pp} & 0_{3\times 3} & 0_{3\times 3}\\ 0_{3\times 3} & 0_{3\times 3} & 0_{3\times 3} & -\beta_{g} & 0_{3\times 3}\\ 0_{3\times 3} & 0_{3\times 3} & 0_{3\times 3} & 0_{3\times 3} & -\beta_{a} \end{array}\right]$$

(6) $$W={\left[\begin{array}{ccccc} w_{g} & w_{a} & 0_{1\times 3} & w_{rg} & w_{ra} \end{array}\right]}^{T}$$

(7) $$H=\left[\begin{array}{ccc} 0_{3\times 6} & I_{3\times 3} & 0_{3\times 6} \end{array}\right]$$

where F is a 15 × 15-dimensional continuous-time state transition matrix; W is a 15-dimensional systematic noise vector; H is a 3 × 15-dimensional measurement update matrix; Z is a 3-dimensional measurement vector of position error; V is a 3-dimensional measurement noise vector; $w_{g}$ and $w_{a}$ denote the noise vector of gyro bias and accelerometer bias, respectively; $w_{rg}$ and $w_{ra}$ denote the noise vector of the first-order Markov process for the gyro and accelerometer, respectively; $\beta_{g}$ and $\beta_{a}$ corresponds to the related time constant of the first-order Markov process. 

Therefore, the discrete EKF system model can be expressed as follows:

(8) $$\left\{\begin{array}{l} {\hat{X}}_{k/k-1}=f\left({\hat{X}}_{k-1}\right)\\ P_{k/k-1}=\Phi_{k/k-1}P_{k-1}\Phi_{k/k-1}^{T}+Q_{k-1}\\ K_{k}=P_{k/k-1}H^{T}{\left(HP_{k/k-1}H^{T}+R_{k}\right)}^{-1}\\ {\hat{X}}_{k}={\hat{X}}_{k/k-1}+K_{k}\left[Z_{k}-H{\hat{X}}_{k/k-1}\right]\\ P_{k}=\left(I_{15\times 15}-K_{k}H\right)P_{k/k-1} \end{array}\right.$$

where $\Phi_{k/k-1}=I_{15\times 15}+F_{k-1}\Delta t$ represents the transition matrix of the system state from time k − 1 to time k; Δt is the sampling interval; ${\hat{X}}_{k/k-1}$ is the system state vector prediction at time k; $f\left(•\right)$ is the state nonlinear function; ${\hat{X}}_{k-1}$ represent the system state estimation vector at time k − 1; $Q_{k-1}$ is the system noise covariance matrix at time k − 1; $R_{k}$ is the measurement noise covariance matrix at time k; $P_{k-1}$ represent the covariance matrix at time k − 1; $P_{k/k-1}$ is the prediction of the covariance matrix at time k; $K_{k}$ is the filter gain matrix at time k; $Z_{k}$ is the system measurement vector at time k.

The state errors are estimated through the EKF algorithm, and the attitude, velocity, and position updated by the SINS feedback are corrected. The equations are as follows:

(9) $$\left\{\begin{array}{l} {\bar{\psi }}_{k,ins}={\tilde{\psi }}_{k,ins}-{\hat{\phi }}_{k}\\ {\bar{v}}_{k,ins}={\tilde{v}}_{k,ins}-\delta {\hat{v}}_{k}\\ {\bar{p}}_{k,ins}={\tilde{p}}_{k,ins}-\delta {\hat{p}}_{k} \end{array}\right.$$

where ${\bar{\psi }}_{k,ins}$, ${\bar{v}}_{k,ins}$ and ${\bar{p}}_{k,ins}$ denote the attitude, velocity, and position of SINS after error correction at time k, respectively; ${\tilde{\psi }}_{k,ins}$, ${\tilde{v}}_{k,ins}$ and ${\tilde{p}}_{k,ins}$ denote the attitude, speed and position vector updated by the SINS at time k, respectively, and include the system errors of the SINS; ${\hat{\phi }}_{k}$, $\delta {\hat{v}}_{k}$ and $\delta {\hat{p}}_{k}$ denote the attitude, velocity, and pose error quantity estimated by the EKF at time k, respectively.

Compensate the state estimation error through feedback to improve the measurement accuracy of the pose of the SINS.

### 2.3. Heading Constraint Model

The GNSS speed angle constraint scheme is used to improve the accuracy of heading measurement. However, the accuracy of the GNSS track angle is related to the speed, especially when the tractor is stationary, the track angle varies randomly between ±180°. Referring to the traditional zero-velocity update [21], when the tractor is in a stationary state and the SINS heading value has not been initialized, set the heading value φ to 0; when the tractor is in a stationary state and the SINS heading initialization has been completed, set the heading value φ to the SINS heading of the tractor at the moment immediately before it entered the stationary state. The observation vector is extended as follows:

(10) $$Z_{k}=\left[\begin{array}{c} {\tilde{p}}_{k,ins}-{\tilde{p}}_{k,gnss}\\ {\tilde{\varphi }}_{k,ins}-\varphi \end{array}\right]$$

where ${\tilde{p}}_{k,gnss}$ represent the position vector updated by the GNSS at time k; ${\tilde{\varphi }}_{k,ins}$ represent the heading updated by the SINS at time k.

A new state observation vector is defined as follows:

(11) $$H=\left[\begin{array}{ccccc} 0_{3\times 2} & 0_{3\times 1} & 0_{3\times 3} & I_{3\times 3} & 0_{3\times 3}\\ 0_{1\times 2} & 1 & 0_{1\times 3} & 0_{1\times 3} & 0_{1\times 3} \end{array}\right]$$

when the tractor is in motion, the GNSS position and track angle are used as observations, and the observation vector is reconstructed as follows:

(12) $$Z_{k}=\left[\begin{array}{c} {\tilde{p}}_{k,ins}-{\tilde{p}}_{k,gnss}\\ {\tilde{\varphi }}_{k,ins}-{\tilde{\varphi }}_{k,gnss} \end{array}\right]$$

where ${\tilde{\varphi }}_{k,gnss}$ represent the heading updated by the GNSS at time k.

According to the GNSS module’s data sheet, the nominal horizontal positioning accuracy is 8 mm, assuming the horizontal distance between two adjacent static positioning points is twice the nominal accuracy (the horizontal distance between two points with a time interval of 0.1 s is 16 mm), the corresponding horizontal speed is 0.16 m/s, which can be regarded as the fluctuation of the static speed measurement error; the nominal speed measurement accuracy is 0.03 m/s, according to the threshold judgment method, the threshold is usually set to 2–5 times the accuracy [22], in view of the vibration disturbance of agricultural tractors, appropriately increased thresholds help minimize misjudgment errors. Therefore, this paper assumed that when the speed is greater than 0.2 m/s, the tractor is in a moving state; conversely, the tractor is in a stationary state.

### 2.4. Adaptive Variance Estimation and Covariance Initialization

The vehicle tractor works in the field, and the amplitude of vehicle body vibration, driving speed, terrain excitation and other factors make the measured values of the GNSS track angle uncertain, making it difficult to calibrate the measurement variance of the track angle. Therefore, the maximum a posteriori estimation algorithm based on Sage–Husa is used to estimate the measurement error variance of the track angle [23]. The measurement error variance is defined as:

(13) $$d_{k}=\left(1-b\right)/\left(1-b^{k+1}\right)$$

(14) $$e_{k}=\delta \varphi_{k}-{\left[HX_{k/k-1}\right]}_{\left(4,1\right)}$$

(15) $$R_{k,\varphi }=\left(1-d_{k}\right)\times R_{k-1,\varphi }+d_{k}\times \left(e_{k}^{2}-{\left[HP_{k/k-1}H^{T}\right]}_{\left(4,4\right)}\right)$$

where $\delta \varphi_{k}={\tilde{\varphi }}_{k,ins}-{\tilde{\varphi }}_{k,gnss}$ represent the heading error at time k; $R_{k,\varphi }$ denote the heading error measurement covariance at time k; $d_{k}$ represent the iteration factor at time k; $e_{k}$ represent the heading error innovation at time k; the bottom-right subscript $\left(i,j\right)$ denotes the i row and j column of the matrix.; b is the forgetting factor, and typically takes values between 0.95 and 0.99 [24]. In this paper, b is set to 0.99.

When the tractor operates at low speed in the field, the randomness of track angle error leads to inaccurate estimation of measurement error variance. This inaccuracy affects the accurate estimation of the heading error angle. Therefore, we propose using the standard deviation of the continuous heading error innovation sequence to replace the single innovation error for conducting an online estimation of the measurement error variance, which is defined as:

(16) $$c_{k}=\sqrt{\frac{1}{m}\sum_{i=k-m+1}^{k}{\left(e_{i}-\frac{1}{m}\sum_{i=k-m+1}^{k}\left(e_{i}\right)\right)}^{2}}+\left|\frac{1}{m}\sum_{i=k-m+1}^{k}\left(e_{i}\right)\right|$$

(17) $$R_{k,\varphi }=\left(1-d_{k}\right)\times R_{k-1,\varphi }+d_{k}\times \left(c_{k}^{2}-{\left[HP_{k/k-1}H^{T}\right]}_{\left(4,4\right)}\right)$$

This method effectively suppresses the influence of large random errors in individual heading measurements on the accurate estimation of the heading measurement variance and ensures a stable estimation of the measurement variance. In this study, m is set to 10.

In addition, the track angle at the instant when the speed first exceeds 0.5 m/s is utilized as the initial heading value of the SINS. At this moment, there is a significant uncertainty error in the initial heading value. Reasonably setting the initial value of the error covariance of the heading error angle in the EKF can accelerate the convergence of the heading error of the SINS.

Therefore, when initializing the heading of the SINS, it is proposed to use the variance statistical value of the track angle variation as the initial value of the covariance of the heading error angle. The equation is defined as follows:

(18) $$P_{k(3,3)}=\frac{1}{N}\sum_{k=1}^{N}{\left(\Delta \varphi_{k,gnss}-\frac{1}{N}\sum_{k=1}^{N}\Delta \varphi_{k,gnss}\right)}^{2}$$

where $\Delta \varphi_{k,gnss}=\left|{\tilde{\varphi }}_{k,gnss}\right|-\left|{\tilde{\varphi }}_{k-1,gnss}\right|$ represents the GNSS track angle variation between time k and time k − 1; N represents the length of the array, which is set to 10 in this paper. 

### 2.5. Experimental Design

**Figure 6:**
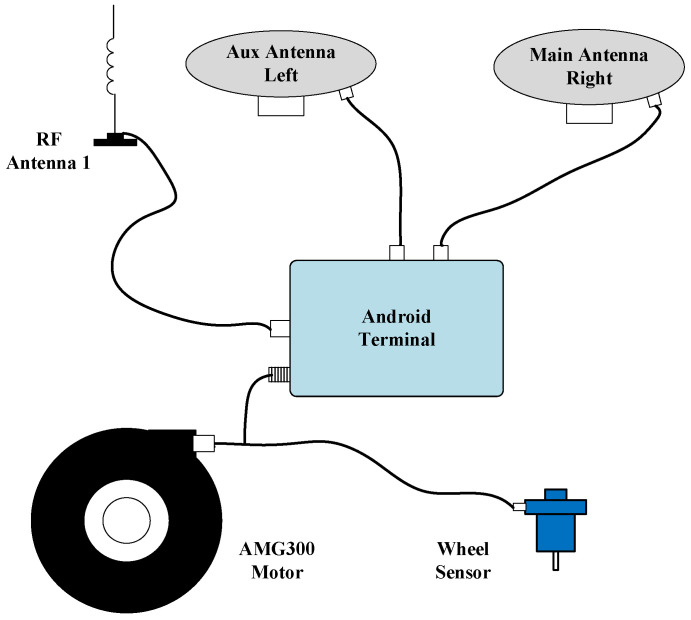
Composition of AMG300 automatic driving system.

Field tests were conducted to validate the proposed algorithm under realistic operating conditions. The experimental setup, shown in Figure 6, includes the AMG300 automatic driving system which ensures controllability of the tractor’s driving path and repeatability of the experiments. The field boundary and navigation path generation method are developed in our previous research [25,26].

**Figure 7:**
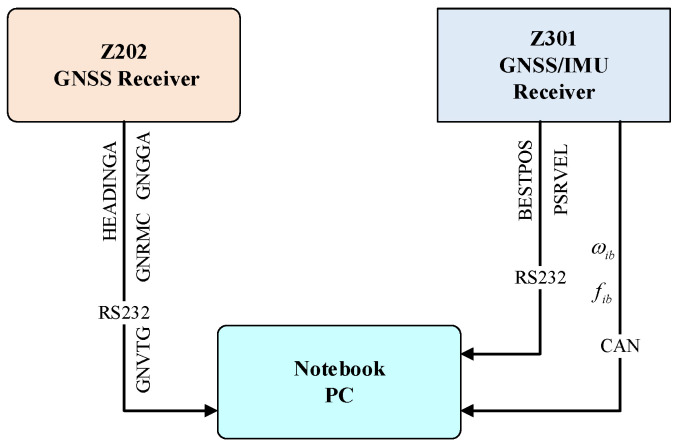
Connection diagram of experimental device.

The connection diagram of experimental devices is illustrated in Figure 7. During experiments, the Z301 receiver transmitted BESTPOS and PSRVEL protocols via serial port at 10 Hz, while simultaneously sending angular velocity and specific force data through the CAN bus at 100 Hz. The Z202 receiver, serving as the reference benchmark, transmitted HEADINGA, GNGGA, GNRMC, and GNVTG protocols via serial port at 10 Hz.

The tests included four scenarios: (1) straight-line driving at 1 m/s from various initial orientations; (2) curved-path driving with fixed steering angles; (3) continuous parallel straight-line tracking; and (4) circular curve navigation. The raw data were processed and analyzed using the method proposed in this study.

## 3. Results

### 3.1. Straight-Driving Test

**Figure 8:**
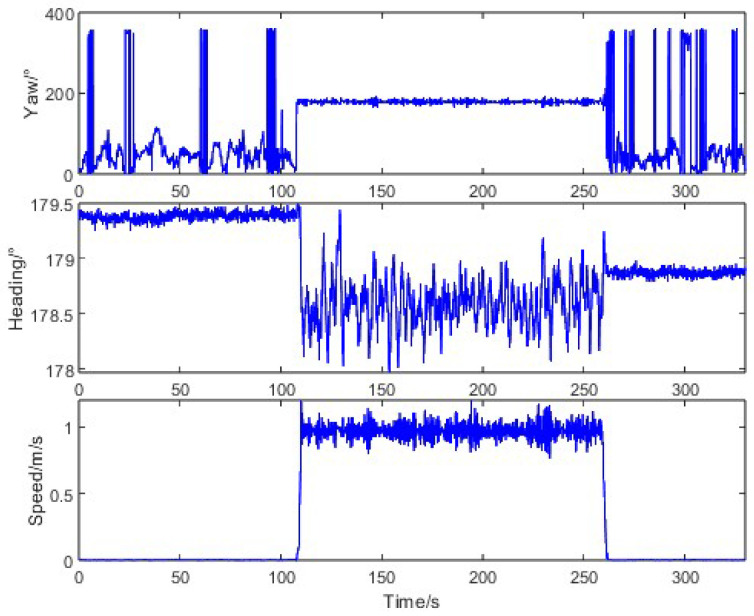
Tractor motion characteristics in straight-driving.

To evaluate the heading measurement performance under typical agricultural operations, we conducted straight-driving tests where the tractor started from stationary states at various orientations and moved along a straight line at a constant speed of 1 m/s. The motion characteristics during this test are illustrated in Figure 8. When the tractor was stationary, the trajectory angle measured by the Z202 receiver varied randomly between 0° and 360°, while the heading angle from the same receiver remained stable with a standard deviation of 0.03°. During straight-line motion, the trajectory angle from the Z301 receiver showed significant fluctuations (standard deviation: 4.41°), whereas the Z202 heading angle maintained consistency (standard deviation: 0.19%), confirming the inadequacy of raw trajectory angles for precision navigation.

**Figure 9:**
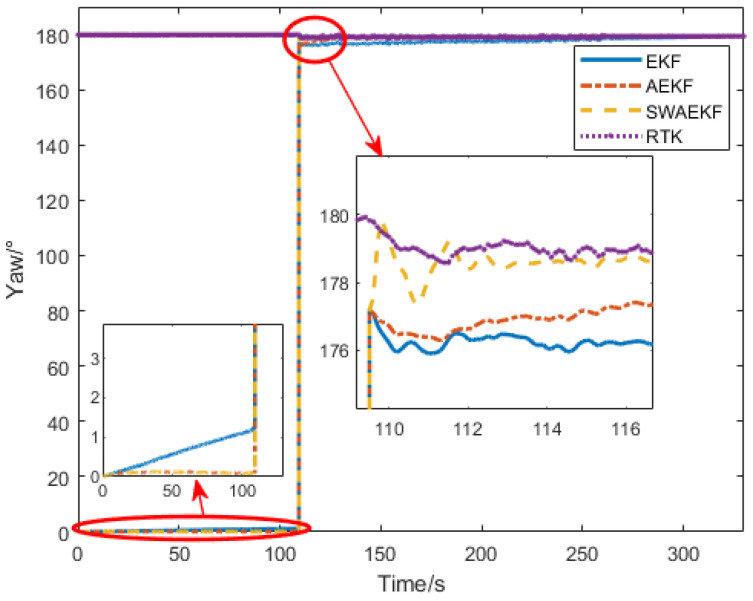
Heading angle comparison in straight-driving.

The heading angle comparisons are presented in Figure 9. Initially, the SINS heading was set to 0° (unknown true heading), while the Z202 reference indicated a southerly direction. The unconstrained EKF algorithm diverged over time, with a drift of 1° at 100 s, due to reliance solely on GNSS position observations. In contrast, the adaptive EKF (AEKF) and sliding-window adaptive EKF (SWAEKF) algorithms utilized static heading constraints during stationary phases, maintaining stability at 0.13°. During motion, both adaptive methods incorporated trajectory angle constraints, enabling convergence.

**Figure 10:**
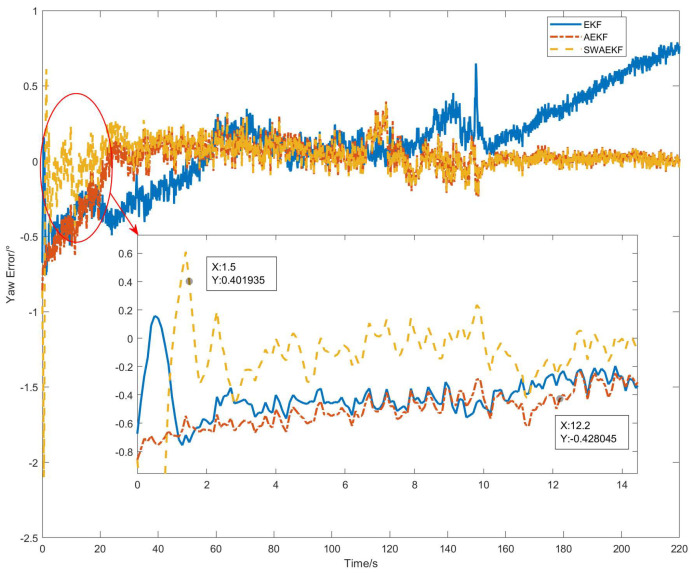
Heading errors in straight-driving.

The heading errors, referenced against the Z202 measurements, are shown in Figure 10. The SWAEKF algorithm achieved the lowest errors, with a standard deviation of 0.09° during motion. Table 2 summarizes the quantitative results from six repeated tests. The SWAEKF reduced the average convergence time to 7.8 s (vs. 34.0 s for AEKF) with an average RMS error of 0.10°, the average convergence time decreases by 77% and the average accuracy is improved by 23%.

### 3.2. Curve-Driving Test

**Figure 11:**
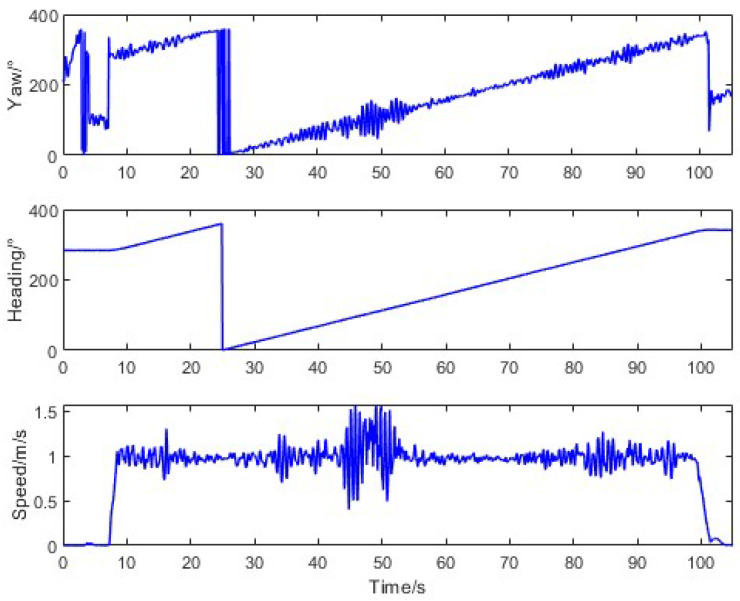
Tractor motion characteristics in curve-driving.

For curved-path scenarios, the tractor was controlled to execute left/right turns at a constant speed of 1 m/s after starting from a stationary state. The motion characteristics, depicted in Figure 11, revealed a substantial error range (−50.08° to 64.92°) between the trajectory angle and the true heading due to non-zero lateral velocity during turns. The average error was 1.90° with a standard deviation of 13.51°, underscoring the challenges of trajectory-based methods in dynamic conditions.

**Figure 12:**
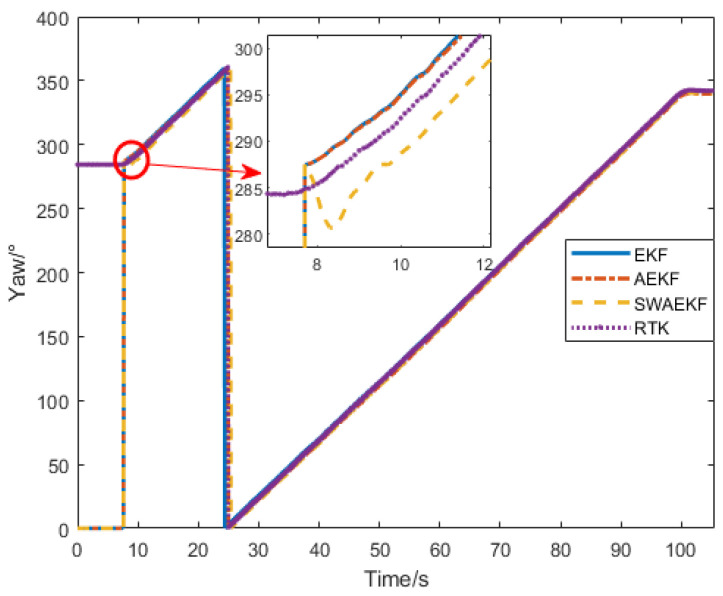
Heading angle comparison in curve-driving.

The heading angle comparisons in Figure 12 show that the SWAEKF algorithm achieved rapid convergence, while the AEKF and unconstrained EKF exhibited slower responses. The initial heading deviation of 2.59° was effectively corrected by SWAEKF’s adaptive constraints.

**Figure 13:**
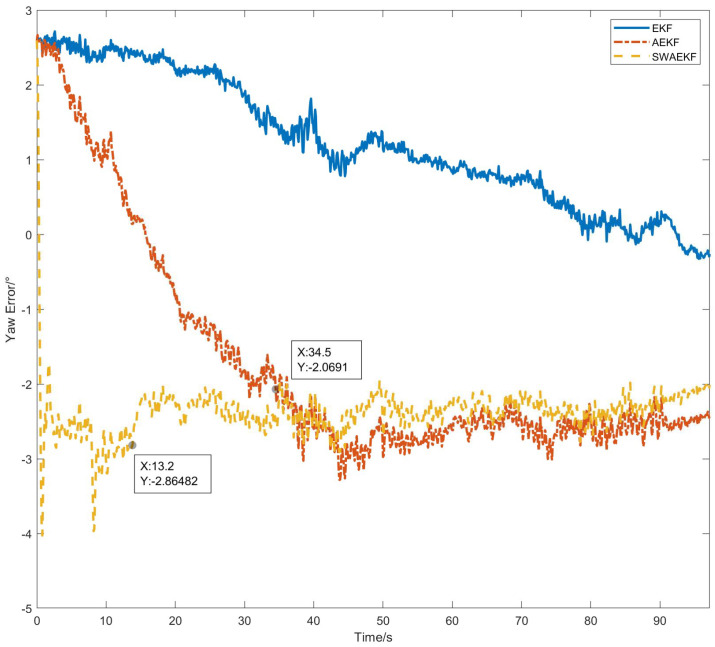
Heading errors in curve-driving.

The heading errors in Figure 13 demonstrate that SWAEKF reduced the convergence time to 13.2 s (vs. 34.5 s for AEKF) and maintained an RMS error of 0.20°. Table 3 consolidates the results, highlighting a 62% improvement in convergence time and a 33% reduction in error standard deviation compared to AEKF.

Compared with the straight-line driving test, the tractor in the curve driving test needs to traverse the agricultural rows originally designed for straight-line operation, which increases the vibration of the tractor during driving. This difference in fluctuation can be observed from the error variation curves of the relative heading of the trajectory in the two groups of tests. From the results, compared with straight-line driving, the curve driving with more severe vibration shows that the standard deviation of the average heading error doubles from 0.1° to 0.2°, and the average convergence time increases from 7.8 s to 12.6 s, with an increase of 62%.

### 3.3. Linear Navigation Test

**Figure 14:**
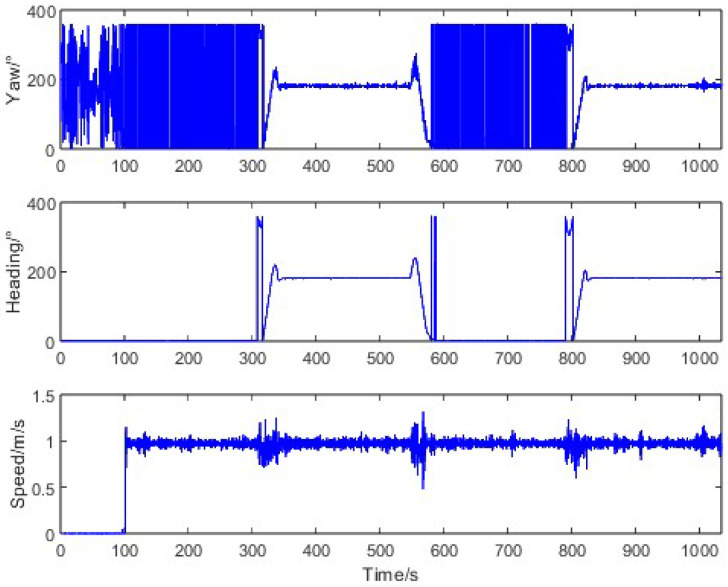
Tractor motion characteristics in linear navigation.

In automated straight-line navigation scenarios, the tractor followed parallel paths using the AMG300 system. As shown in Figure 14, the trajectory-heading error varied between −22.12° and 17.80°, with standard deviations of 2.29–3.17° across segments, indicating significant noise under operational conditions.

**Figure 15:**
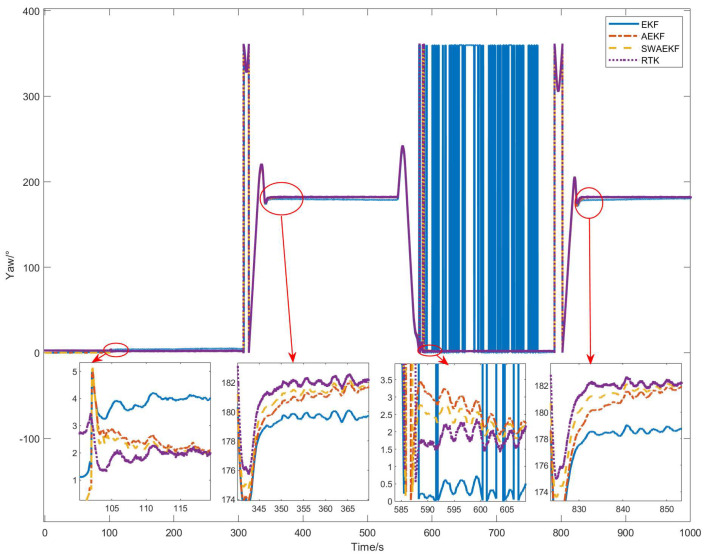
Heading angle comparison in linear navigation.

The heading angle comparisons in Figure 15 reveal that the SWAEKF converged within 6.2 s after initialization, while the AEKF required 8.3 s. The unconstrained EKF diverged during straight-line segments but converged temporarily during turns due to velocity changes.

**Figure 16:**
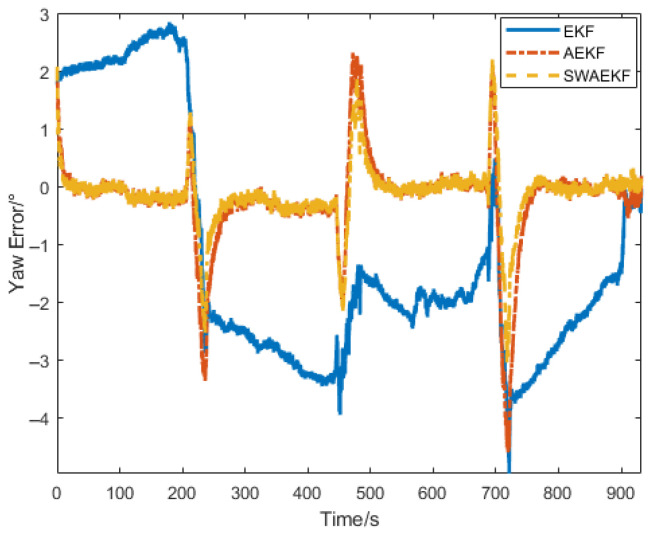
Heading errors in linear navigation.

The heading errors in Figure 16 and Table 4 confirm that the SWAEKF achieved an RMS error of 0.54° during linear navigation (vs. 0.81° for AEKF), the average accuracy is improved by 33%. The heading errors within ±0.5° during straight-line segments, with rapid re-convergence after turns.

### 3.4. Curve Navigation Test

**Figure 17:**
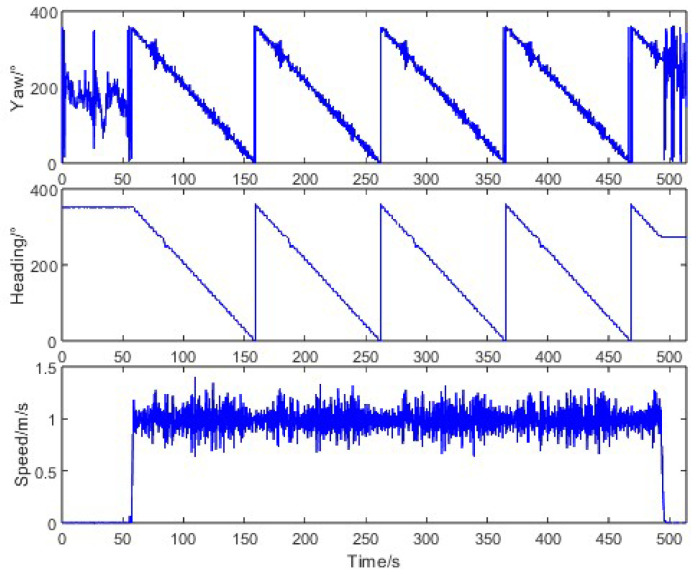
Tractor motion characteristics in curve navigation.

For circular path tracking, the tractor navigated a curve divided into 3-m straight segments via the AMG300 system. The motion characteristics in Figure 17 show trajectory-heading errors ranging from −41.42° to 38.36° (mean: −1.38°, std: 7.74°), with large fluctuations during segment transitions.

**Figure 18:**
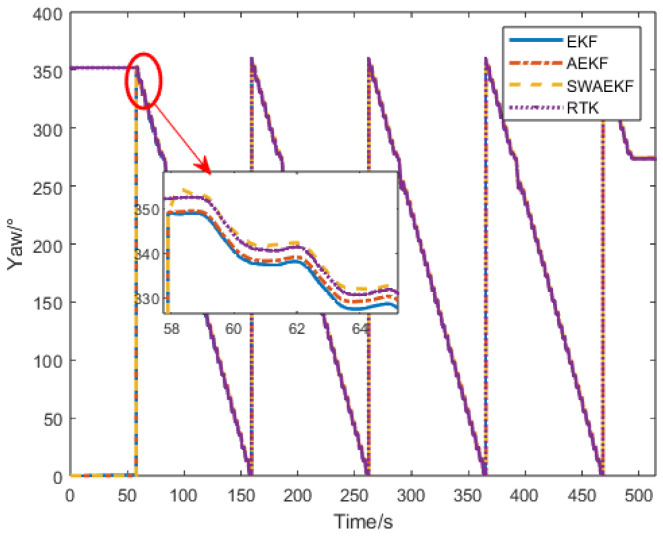
Heading angle comparison in curve navigation.

**Figure 19:**
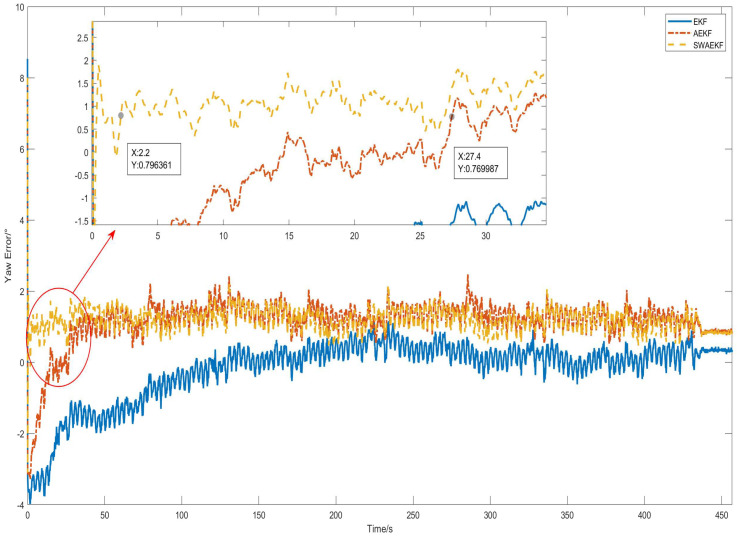
Heading error comparison in curve navigation.

The heading error comparisons in Figure 18 and Figure 19 and Table 5 demonstrate that the SWAEKF achieved an RMS error of 0.28° (vs. 0.65° for AEKF), the average accuracy is improved by 57%. The algorithm showed stability despite sudden heading changes during segment switches.

Compared with the straight-line navigation state, during curved-path navigation, the trajectory constraint method causes the fusion-estimated heading value to exhibit a deviation relative to the heading value measured by dual-antenna GNSS.

## 4. Discussion

**Figure 20:**
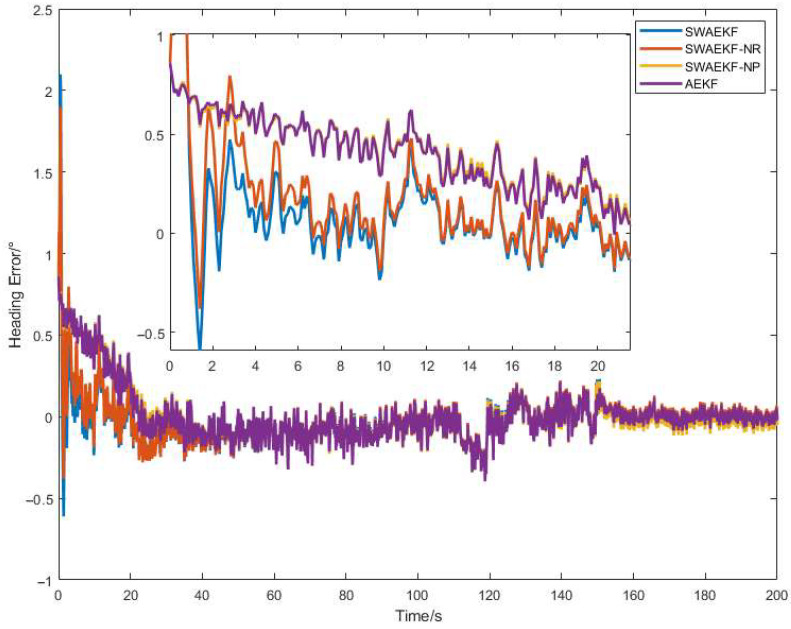
Factor ablation comparative analysis.

The experimental results demonstrate that the proposed SWAEKF algorithm significantly outperforms both the conventional EKF and AEKF across all test scenarios. The SWAEKF algorithm incorporates three key improvements: (1) Distinct constraint conditions are applied to the dynamic and static operating states of the tractor, the objective is to achieve accurate heading tracking under dynamic conditions and stable heading maintenance under static conditions, and this design is independent of the convergence rate of the initial heading. (2) A novel calculation method for the track angle error variance (denoted as SWAEKF-NP) is adopted, the purpose is to enhance the heading accuracy of the fusion-based measurement by adaptively increasing the track angle error variance, though it slightly reduces the convergence rate of the initial heading error. (3) During heading initialization, the heading error covariance is updated using the track angle fluctuation variance (denoted as SWAEKF-NR). This aims to accelerate the heading convergence rate of the fusion-based measurement. The comprehensive full-factor comparative analysis was conducted on the second and third improvements, with the results presented in Figure 20.

As evidenced in Table 6, the SWAEKF achieved an average convergence time of 7.8 s in straight-line tests, representing a 77% improvement over the AEKF (34.0 s). This accelerated convergence can be attributed to the adaptive covariance initialization strategy, which effectively captures the uncertainty in initial heading estimation. While the sliding-window-based variance estimation method has no significant effect on the heading convergence time.

**Figure 21:**
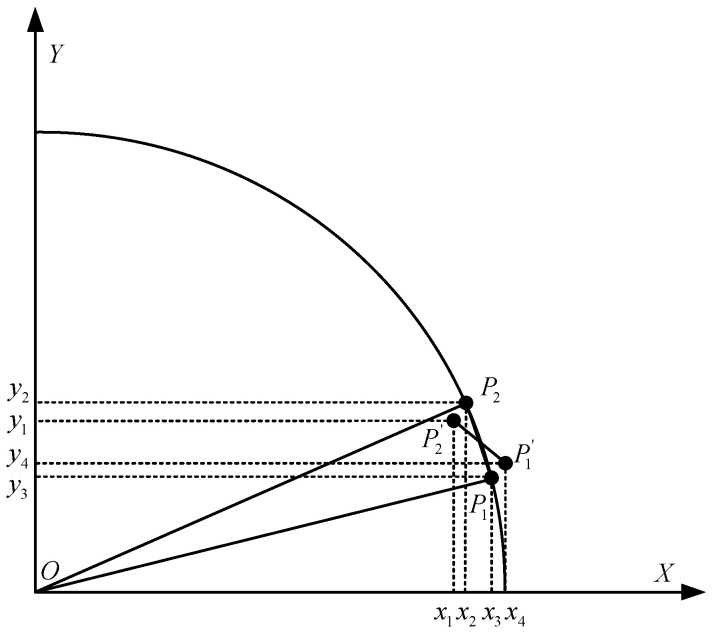
Analysis of the tractor track angle fluctuation.

Our comparative analysis of straight-line and circular-curve driving modes reveals significantly higher heading accuracy in straight-line scenarios. This performance discrepancy primarily stems from the inherent challenges in circular navigation, where the tractor’s trajectory is segmented into successive straight-line segments by the navigation control system. While the ideal trajectory assumes smooth curvature (Figure 21), practical implementation introduces discrete transitions between segments. At the operational speed of 1 m/s, the theoretical distance between trajectory points is 10 cm; however, GNSS-RTK positioning errors combined with dynamic body attitude variations cause actual point-to-point distances to fluctuate around this ideal value. These fluctuations induce substantial trajectory angle errors during segment transitions, ultimately compromising heading estimation accuracy in curved paths.

Furthermore, the tractor’s dynamic centroid sideslip angle introduces additional complexity to heading estimation. This parameter is influenced by multiple factors including soil surface structure, load distribution, and tire–terrain interaction characteristics in farmland environments. Our trajectory-constrained heading measurement method essentially captures the tractor’s velocity direction, which inherently differs from the actual vehicle heading direction due to the presence of sideslip angles. The track angle provided by the GNSS module is essentially the angle between the tractor’s horizontal displacement vector and the true north direction. This horizontal displacement vector is constructed based on the horizontal positioning points output at consecutive moments, and the aforementioned angle corresponds to the track angle. For a tractor, the track angle reflects the instantaneous velocity direction of the vehicle. When skidding occurs, the theoretical value of the angle between the tractor’s heading and its theoretical track angle is expressed as follows:

(19) $$\theta =\arctan(\frac{v_{y}}{v_{x}})$$

where $v_{y}$ represents the vertical speed of the tractor, $v_{x}$ represents the longitudinal speed of the tractor.

It can be concluded that the greater the vertical velocity component of the tractor during travel, the larger the difference between the heading value obtained by fusion estimation and the heading of the tractor’s central axis. When the tractor travels along a circular curve, there exists a systematic deviation between the heading measured by fusion and the heading relative to the carrier. For non-four-wheel steering tractors, displacement is inevitable during travel, so the speed threshold method can be used to determine their stationary state. For tractors capable of in-place steering, the speed threshold-based judgment method is invalid.

The velocity threshold approach employed for heading initialization, while effective, presents opportunities for further refinement. Our use of 0.5 m/s as the threshold for SINS heading initialization represents a practical compromise between early convergence and measurement reliability. However, analysis of the straight-line tests reveals that initialization accuracy is highly dependent on the initial heading deviation magnitude. Cases with smaller initial deviations (e.g., 0.86°) achieved convergence within 1.5 s, while larger deviations (8.03°) required up to 15.6 s for convergence. This dependency suggests that adaptive thresholding based on trajectory quality metrics could enhance initialization robustness, particularly under challenging field conditions where GNSS signal quality varies.

When compared to existing heading estimation approaches, our SWAEKF method demonstrates distinct advantages in agricultural applications. Unlike optimization-based alignment methods that require specific vehicle maneuvers [6,7,8], our trajectory-constrained approach maintains effectiveness during the straight-line operations predominant in field work. Compared to non-holonomic constraint methods [13,14] that assume ideal rolling conditions, our adaptive variance estimation mechanism better accommodates the real-world terrain variations encountered in farmland operations. The 62% convergence time improvement over conventional AEKF in curved-path tests particularly highlights the efficacy of our sliding-window innovation processing in handling GNSS measurement uncertainties.

Several limitations of the current study warrant attention in future research. The performance degradation observed during circular curve navigation indicates that sideslip angle dynamics are not fully addressed by our current model. Additionally, the fixed velocity threshold for heading initialization may not optimally adapt to varying field conditions. Future work should investigate real-time sideslip angle estimation techniques that integrate additional sensor modalities or exploit kinematic models. Furthermore, adaptive thresholding strategies that dynamically adjust based on GNSS quality indicators could enhance initialization robustness. The development of ultra-low-speed heading estimation methods remains particularly important for precision agricultural applications requiring precise maneuverability.

## 5. Conclusions

This study has developed and validated an adaptive trajectory-constrained heading estimation method that effectively addresses the challenges of single-antenna GNSS/SINS integration for tractors operating in low-dynamic farmland environments. The proposed SWAEKF algorithm incorporates three key innovations: a switching observation model that adapts to vehicle motion states, an enhanced Sage–Husa adaptive variance estimation mechanism with sliding-window processing, and a covariance initialization strategy based on trajectory angle statistics. Experimental results demonstrate that our method achieves a 23% improvement in heading accuracy and reduces convergence time by 62% compared to conventional adaptive EKF approaches. The algorithm maintains RMS heading errors below 0.15° in straight-line tracking and 0.25° in automated curve navigation, meeting the precision requirements for autonomous navigation of small-to-medium tractors. Future research will focus on integrating dynamic sideslip compensation and developing adaptive initialization protocols to further enhance performance in challenging operating conditions.

## Figures and Tables

**Table 1:** Specifications of the key equipment.

Component	Parameters
GNSS Receiver (Z301)	UM982 module (Ver. 11826, Unicore Communications, Inc., Beijing, China), RTK horizontal accuracy: 8 mm + 1 ppm RMS, update rate: 10 Hz, reliability: 99.9%, velocity accuracy (RMS): 0.03 m/s
IMU (FSS-IMU614E-P)	Gyro bias stability: 4.5°/h, accelerometer range: ±6 g, update rate: 100 Hz
Reference (Z202)	Heading accuracy: 0.1°/1 m baseline, dual-antenna configuration

**Table 2:** Accuracy and convergence time of heading under straight-driving test.

Index	AEKF				SWAEKF			
	Mean/(°)	Std/(°)	Max/(°)	Time/s	Mean/(°)	Std/(°)	Max/(°)	Time/s
1	0.06	0.09	0.40	12.2	0.06	0.09	0.38	1.5
2	0.02	0.11	0.45	60.3	0.14	0.09	0.44	15.6
3	0.17	0.16	0.47	48.1	0.05	0.11	0.29	9.8
4	−0.04	0.14	0.50	25.1	−0.02	0.09	0.43	8.5
5	0.20	0.18	0.63	17.8	0.21	0.11	0.50	2.3
6	0.04	0.11	0.45	40.6	0.06	0.10	0.33	8.9
Mean	0.08	0.13	0.48	34.0	0.08	0.10	0.40	7.8

**Table 3:** Accuracy and convergence time of heading under curve-driving test.

Index	AEKF				SWAEKF			
	Mean/(°)	Std/(°)	Max/(°)	Time/s	Mean/(°)	Std/(°)	Max/(°)	Time/s
1	−2.38	0.27	3.28	34.5	−2.41	0.16	2.91	13.2
2	−2.10	0.25	2.60	24.5	−2.27	0.20	2.88	13.3
3	1.98	0.48	2.86	41.1	2.03	0.22	2.48	11.3
4	2.51	0.24	3.19	39.1	2.60	0.15	3.03	11.5
5	1.94	0.31	2.51	34.3	1.97	0.25	2.54	12.9
6	−2.13	0.23	2.72	27.2	−2.22	0.22	2.77	13.1
Mean	−0.03	0.30	2.86	33.5	−0.05	0.20	2.77	12.6

**Table 4:** Accuracy heading in linear navigation.

Indicators	AEKF	SWAEKF
Mean (°)	−0.22	−0.13
Std (°)	0.81	0.54

**Table 5:** Accuracy heading in curve navigation.

Indicators	AEKF	SWAEKF
Mean (°)	1.16	1.23
Std (°)	0.65	0.28

**Table 6:** Comparison of Heading Convergence Time.

Index	SWAEKF/s	SWAEKF-NR/s	SWAEKF-NP/s	AEKF/s
1	1.5	3.5	12.2	12.2
2	15.6	15.5	75.3	60.3
3	9.8	9.8	42.3	48.1
4	2.3	2.3	18.6	17.8
5	8.5	7.5	28.4	25.1
6	8.9	8.9	23.4	40.6
mean	7.8	7.9	33.4	34.0

## Data Availability

Data available on request due to restrictions on privacy.

## Abbreviations

The following abbreviations and nomenclature are used in this manuscript:

**Abbreviations**	
GNSS	Global Navigation Satellite System
SINS/INS	Strapdown Inertial Navigation System
IMU	Inertial Measurement Unit
MEMS	Micro-Electro-Mechanical Systems
EKF	Extended Kalman Filter
AEKF	Adaptive Extended Kalman Filter
SWAEKF	Sliding-Window Adaptive Extended Kalman Filter
RTK	Real-Time Kinematic
RMS	Root Mean Square
NHC	Non-Holonomic Constraints
**Nomenclature**	
^˜^	variable contains errors
^^^	variable is estimated
^¯^	variable is corrected
×	skew symmetric matrix operator
Superscript	
b	variable in the tractor body coordinates (b-frame)
n	variable in the navigation coordinates (n-frame)
T	transpose operator for matrices or vectors
Subscript	
k	time k
k/k−1	prediction at time k from the k-1 time
k,ins	INS date at time k
k,gnss	GNSS date at time k
k,φ	yaw data at time k
Variables	
$C_{b}^{n}$	attitude matrix from the b-frame to the n-frame
$\omega_{ib}^{b}$	angular rate vector of gyro (measured in the b-frame)
$\omega_{ie}^{n}$	Earth’s angular rate vector (measured in the n-frame)
$\omega_{en}^{n}$	angular rate vector of the n-frame relative to the e-frame (measured in the n-frame)
v	velocity vector of SINS
$f_{\mathrm{sf}}^{b}$	specific force vector of accelerometer (measured in the b-frame)
g	gravity acceleration vector
p	position vector of SINS
ϕ	attitude error vector
δv	velocity error vector
δp	position error vector
ε	gyro bias
𝛻	accelerometer bias
$M_{aa}$	transfer matrix of attitude to the differential of attitude
$M_{av}$	transfer matrix of velocity to the differential of attitude
$M_{ap}$	transfer matrix of position to the differential of attitude
$M_{va}$	transfer matrix of attitude to the differential of velocity
$M_{vv}$	transfer matrix of velocity to the differential of velocity
$M_{vp}$	transfer matrix of position to the differential of velocity
$M_{pv}$	transfer matrix of velocity to the differential of position
$M_{pp}$	transfer matrix of position to the differential of position
X	state variables of SINS errors
F	continuous-time state transition matrix of SINS errors
W	SINS noise vector
Z	measurement vector of position error
H	measurement matrix of position error
V	noise vector of position error
$w_{g}$	noise vector of gyro bias
$w_{a}$	noise vector of accelerometer bias
$w_{rg}$	noise vector of the first-order Markov process for the gyro
$w_{ra}$	noise vector of the first-order Markov process for the accelerometer
$\beta_{g}$	time constant matrix the first-order Markov process for the gyro
$\beta_{a}$	time constant matrix the first-order Markov process for the accelerometer
$f\left(•\right)$	state nonlinear function of Kalman Filter
Φ	system state transition matrix of Kalman Filter
P	covariance matrix of Kalman Filter
K	gain matrix of Kalman Filter
Q	system noise covariance matrix
R	measurement noise covariance matrix
ψ	attitude vector
φ	yaw angle
d	iteration factor
e	heading error innovation
c	precision factor of heading error
$R_{k,\varphi }$	heading error measurement covariance at time k

## References

[1] An G., Yu C., Du J., Yin X., Ni Y., Jin C. (2024). Development of the Electric Automatic Steering System for Agricultural Vehicles. Int. J. Agric. Biol. Eng..

[2] Rakun J., Pantano M., Lepej P., Lakota M. (2022). Sensor Fusion-Based Approach for the Field Robot Localization on Rovitis 4.0 Vineyard Robot. Int. J. Agric. Biol. Eng..

[3] Sultan Mahmud M., Zahid A., He L., Choi D., Krawczyk G., Zhu H. (2021). LiDAR-Sensed Tree Canopy Correction in Uneven Terrain Conditions Using a Sensor Fusion Approach for Precision Sprayers. Comput. Electron. Agric..

[4] Tayebi A., Gómez J., Fernández M., Sáez de Adana F., Gutiérrez O. (2021). Low-Cost Experimental Application of Real-Time Kinematic Positioning for Increasing the Benefits in Cereal Crops. Int. J. Agric. Biol. Eng..

[5] Zhang Q., Chen Q., Xu Z., Zhang T., Niu X. (2021). Evaluating the navigation performance of multi-information integration based on low-end inertial sensors for precision agriculture. Precis. Agric..

[6] Mohammadkarimi H., Mozafari S., Alizadeh M.H. (2024). Analytical Quaternion-Based Bias Estimation Algorithm for Fast and Accurate Stationary Gyro-Compassing. Sci. Rep..

[7] Chen Y., Li W., Wang Y. (2021). A Robust Adaptive Indirect In-Motion Coarse Alignment Method for GPS/SINS Integrated Navigation System. Measurement.

[8] Wu Q., Chai H., Xiang M., Zhang Y., Zhang F., Feng X. (2024). Rapid in-motion heading alignment using time-differenced carrier phases from a single GNSS antenna and a low-grade imu. Meas. Sci. Technol..

[9] Lu Z., Li J., Zhang X., Feng K., Wei X., Zhang D., Mi J., Liu Y. (2020). A New In-Flight Alignment Method with an Application to the Low-Cost SINS/GPS Integrated Navigation System. Sensors.

[10] Chen Q., Lin H., Guo R., Niu X. (2020). Rapid and Accurate Initial Alignment of the Low-Cost MEMS IMU Chip Dedicated for Tilted RTK Receiver. GPS Solut..

[11] Chen Q., Lin H., Kuang J., Luo Y., Niu X. (2023). Rapid Initial Heading Alignment for MEMS Land Vehicular GNSS/INS Navigation System. IEEE Sens. J..

[12] Mao Y., Sun R., Wang J., Cheng Q., Kiong L.C., Ochieng W.Y. (2022). New Time-Differenced Carrier Phase Approach to GNSS/INS Integration. GPS Solut..

[13] Zhang T., Liu S., Chen Q., Feng X., Niu X. (2022). Carrier-Phase-Based Initial Heading Alignment for Land Vehicular MEMS GNSS/INS Navigation System. IEEE Trans. Instrum. Meas..

[14] Fu Q., Liu Y., Liu Z., Li S., Guan B. (2018). Autonomous In-Motion Alignment for Land Vehicle Strapdown Inertial Navigation System without the Aid of External Sensors. J. Navig..

[15] Li H., Liu Z., Li C., Zheng Y., Tong S., Chen S., Gou W. (2024). Non-Holonomic Constraint-Assisted GNSS/SINS Tight Integration Navigation Method Based on a Left-Invariant Extended Kalman Filter. Meas. Sci. Technol..

[16] Fossen S., Fossen T.I. (2021). Five-State Extended Kalman Filter for Estimation of Speed over Ground (SOG), Course over Ground (COG) and Course Rate of Unmanned Surface Vehicles (USVs): Experimental Results. Sensors.

[17] Zhang Q., Li S., Xu Z., Niu X. (2020). Velocity-Based Optimization-Based Alignment (VBOBA) of Low-End MEMS IMU/GNSS for Low Dynamic Applications. IEEE Sens. J..

[18] Li S., Zhang M., Ji Y., Zhang Z., Cao R., Chen B., Li H., Yin Y. (2021). Agricultural Machinery GNSS/IMU-Integrated Navigation Based on Fuzzy Adaptive Finite Impulse Response Kalman Filtering Algorithm. Comput. Electron. Agric..

[19] Chen K., Chang G., Chen C. (2021). GINav: A MATLAB-Based Software for the Data Processing and Analysis of a GNSS/INS Integrated Navigation System. GPS Solut..

[20] Hu Q., Fan Z., Zhang X., Sun N., Li X., Qiu Q. (2025). Robust Localization and Tracking Control of High-Clearance Robot System Servicing High-Throughput Wheat Phenotyping. Comput. Electron. Agric..

[21] Weng J., Liu J., Jiao M., Kou K. (2020). Analysis and On-Line Compensation of Gravity Disturbance in a High-Precision Inertial Navigation System. GPS Solut..

[22] Li L., Pan Y., Lee J.K., Ren C., Liu Y., Grejner-Brzezinska D.A., Toth C.K. (2012). Cart-Mounted Geolocation System for Unexploded Ordnance with Adaptive ZUPT Assistance. IEEE Trans. Instrum. Meas..

[23] Fan Y., Qiao S., Wang G., Zhang H. (2024). An Improved Sage-Husa Variational Robust Adaptive Kalman Filter With Uncertain Noise Covariances. IEEE Sens. J..

[24] Tseng C.H., Wu J.Y. (2024). SHAKF-PU: Sage–Husa Adaptive Kalman Filtering-Based Pedestrian Characteristic Parameter Update Mechanism for Enhancing Step Length Estimation in Pedestrian Dead Reckoning. Appl. Bionics Biomech..

[25] Wang H., Noguchi N. (2018). Adaptive turning control for an agricultural robot tractor. Int. J. Agric. Biol. Eng..

[26] Wang H., Ma Z.F., Ren Y.X., Du S.Q., Lu H., Shang Y., Hu S., Zhang G., Meng Z., Wen C. (2024). Interactive image segmentation based field boundary perception method and software for autonomous agricultural machinery path planning. Comput. Electron. Agric..

